# Esophagitis Dissecans Superficialis: Unveiling the Enigmatic Entity of Esophageal Mucosal Sloughing

**DOI:** 10.7759/cureus.43549

**Published:** 2023-08-15

**Authors:** Abeer Qasim, Abhilasha Jyala, Haider Ghazanfar, Aam Baqui, Harish Patel

**Affiliations:** 1 Internal Medicine, BronxCare Health System, New York, USA; 2 Pathology, BronxCare Health System, New York, USA

**Keywords:** mucosal disintegration, rare esophageal condition, superficial esophageal erosion, shedding esophageal lining, sloughing esophageal mucosa

## Abstract

Esophagitis dissecans superficialis (EDS), formerly referred to as sloughing esophagitis, is a degenerative condition affecting the squamous epithelium. EDS is known to be a benign condition that resolves on its own. The exact etiology of EDS remains unclear, although associations with medications like bisphosphonates or nonsteroidal anti-inflammatory drugs, skin conditions, heavy smoking, and physical trauma have been reported. The clinical manifestations exhibit a wide range, encompassing both incidental findings and symptomatic presentations related to the esophagus. Here we present an interesting case of a middle-aged female patient with dysphagia who underwent early esophagogastroduodenoscopy (EGD) for timely identification and treatment of EDS, emphasizing the significance of early detection and management.

## Introduction

Esophagitis dissecans superficialis (EDS), also known as sloughing esophagitis or desquamative esophagitis, is a rare and intriguing condition that is characterized by the sloughing of large fragments of the esophageal mucosal lining [1]. According to a retrospective analysis of 21,497 cases of esophagogastroduodenoscopy (EGD) revealed an incidence of EDS at 0.03% [2]. EDS is a non-threatening condition that typically resolves by itself. The entity known as sloughing esophagitis was initially documented in 1892. However, it remains an overlooked condition that needs more reporting in medical literature [3]. The symptoms of EDS can vary and may not always be apparent; reported symptoms include dysphagia, dyspepsia, noncardiac chest pain, and odynophagia [4]. Diagnosing esophagitis dissecans involves a combination of clinical evaluation, endoscopic examination, histopathological analysis, and exclusion of other possible causes. EGD is a crucial diagnostic procedure for evaluating esophagitis dissecans [5]. There is no established standard treatment for EDS since many cases resolve spontaneously without long-term complications. This case report highlights the challenges associated with diagnosing EDS due to its rarity and potential resemblance to other esophageal disorders.

## Case presentation

A 62-year-old female patient with a past medical history of hypertension presented to the emergency department (ED) with a complaint of severe epigastric pain. The pain was described as sharp, non-radiating, accompanied by nausea and non-bilious, non-bloody vomiting that persisted two days prior to the presentation. The patient was unable to tolerate oral intake. She denied fever, chills, or any episodes of hematemesis or melena. The patient had a prior abdominal surgery, details of which are not known. The patient occasionally consumed wine but denied smoking or using illicit drugs. The patient denied taking any medications at home. Family history was not significant for gastrointestinal (GI) malignancy. Upon arrival at the ED, the patient was hemodynamically stable. The laboratory results revealed the presence of anemia, leukocytosis, transaminitis, hypocalcemia (7.9 mg/dL) (normal 8.5-10.5mg/dL), and increased lipase (1921 U/L) (normal <=61 U/L) and C-reactive protein (CRP) levels. Also, the patient was found to have an elevated antinuclear antibody (ANA) titer of 1:640. Detailed laboratory findings are discussed in Table 1.

**Table 1:** Lab findings HGB: hemoglobin, WBC: white blood count, RDW: red cell distribution width, AST: aspartate aminotransferase, INR: international normalized ratio, PT: prothrombin time, ANA: antinuclear antibody, SS: Sjögren's antibodies, IgG: immunoglobulin G

Test Name	Result	Reference Range
HGB	10.2g/dL	12.0-16.0 g/dL
Hematocrit, Whole Blood	30.7%	42.0-51.0%
Eosinophil %	2.2%	<= 5.0%
WBC Count	13.7k/ul	4.8-10.8 k/ul
RDW	14.1%	10.5-14.5%
Platelet	291 k/ul	150-400 k/ul
Neutro %	84.3%	40.0-70.0%
Lymphocyte Count	1.4 k/ul	1.0-4.8 k/ul
C Reactive Protein, Serum	161.57mg/L	<= 5.00 mg/L
Triglycerides, Serum	83 mg/dL	56-150 mg/dL
Alanine Aminotransferase, Serum	339units/L	5-40 unit/L
AST	503 unit/L/L	9-36 unit/L/L
Lactic Acid Level	0.9 mmoles/L	0.5-1.6 mmoles/L
Bilirubin, Serum Direct - Conjugated	0.3 mg/dL	0.2-1.1 mg/dL
Bilirubin, Serum Total	0.2 md/dL	0.0-0.3 mg/dL
Total Protein Serum	7.0 g/dL	5.8-8.3 g/dL
Gamma Glutamyl Transferase, Serum	162units/L	8-54 unit/L
Acetaminophen Level, Serum	<5.0ug/L	10.0-30.0 ug/mL
Alanine Aminotransferase, Serum	188unit/L	5-40 unit/L
Hepatitis A Total Antibody	Reactive	-
Hepatitis B Core IgM Antibody	Non Reactive	-
Hepatitis B Surface Antibody	Non Reactive	-
HCV ANTIBODY-EIA	Non Reactive	-
INR	0.98	0.85-1.14
Anticoagulant	u	-
PT	11.5 seconds	9.9-13.3 seconds
Creatine Kinase, Serum	98 unit/L	20-200 unit/L
Ferritin, Serum	53.3 ng/L	13.0-150.0 ng/mL
Unsaturated Iron Binding Capacity	298 ug/dL	112-346 ug/dL
Iron, Serum	<5ug/dL	65-175 ug/dL
Blood Urea Nitrogen, Serum	17.0 mg/dL	6.0-20.0 mg/dL
Creatinine, Serum	7.9(L) 8.5-10.5 mg/dL	8.5-10.5 mg/dL
ANA Titer	1:640	Negative
ANA	Positive	Negative
IgG 4 subclass	14.8mg/dL	4-86mg/dL
Antibody to Antiscleroderma-70	<1.0 negative	<1.0 negative
SS-A Ab	<1.0 negative	<1.0 negative
SS-B Ab	<1.0 negative	<1.0 negative
Anti-DNA Ab	8 IU/mL	≤4 IU/mL negative
Total hemolytic complement assay	>60U/mL	31-60U/mL

**Figure 1:**
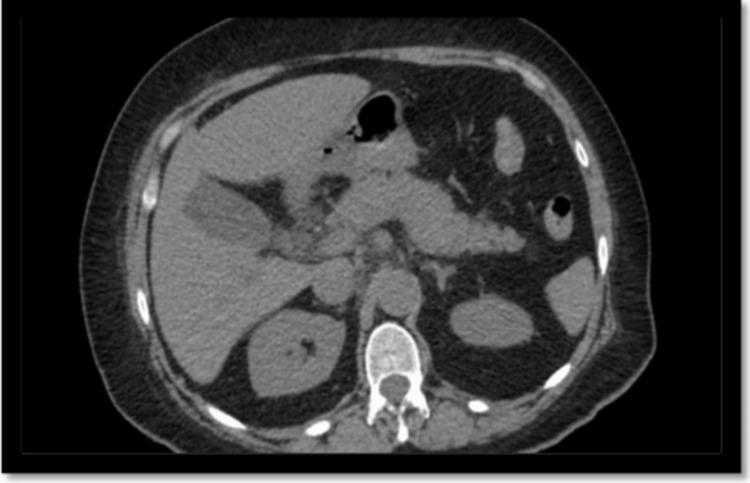
CT scan of the abdomen (with and without contrast): Mild fatty infiltration of liver. Partially calcified gallbladder wall. Mild perihepatic ascites and mild pericholecystic fluid. Mild diffuse peripancreatic stranding compatible with acute pancreatitis. No evidence of pseudocyst or abscess collections. Duodenal diverticulum. CT: computed tomography

On physical examination, the patient was noted to have tenderness in the epigastrium only, the rest of the examination was benign. The patient underwent an abdomen ultrasound; however, the examination was limited due to the presence of overlying bowel gas, hindering visibility. Mild hepatomegaly and diffuse increased echogenicity of hepatic parenchyma were observed, which could be attributed to mild fatty infiltration or hepatocellular disease. The patient underwent a computed tomography (CT) scan of the abdomen revealed mild fatty infiltration of the liver, a partially calcified gallbladder wall, as well as mild perihepatic ascites and mild pericholecystic fluid (Figure 1).

**Figure 2:**
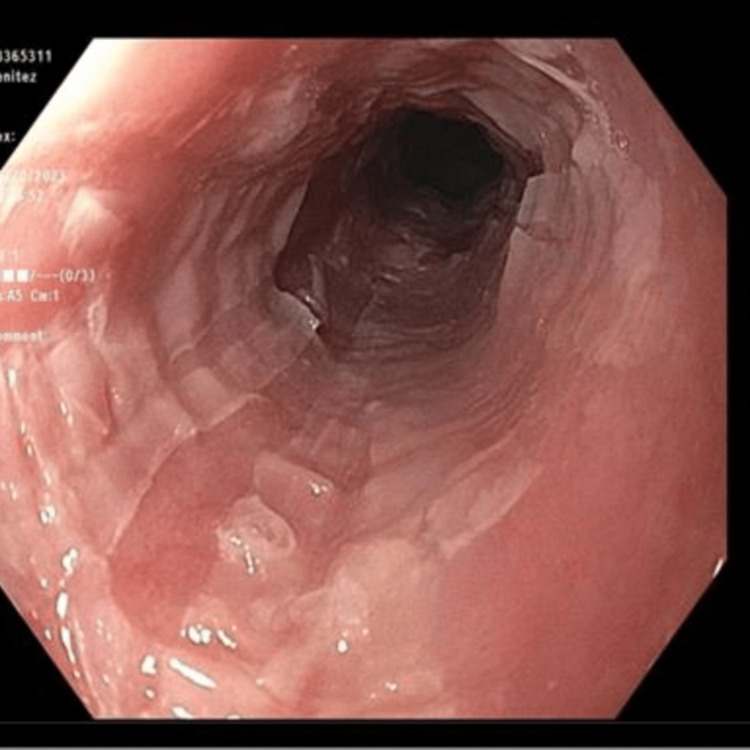
Esophagogastroduodenoscopy (EGD): columns of mucosal tissue are found in the lower portion of the esophagus.

Considering the initial diagnosis of acute pancreatitis, the patient was started on intravenous fluid hydration and anti-emetics, and medications were given for pain control. For the elevated ANA titer, a further workup for autoimmune diseases was done. The patient was found to have an elevated level of anti-double-stranded DNA (dsDNA) along with elevated levels of total hemolytic complement assay CH 50 (Table 1). CH 50 levels could be elevated in the setting of acute inflammation from acute pancreatitis; hence the decision was made to follow up as an outpatient for further evaluation of undiagnosed systemic lupus erythematosus (SLE). The patient experienced gradual improvement and was able to tolerate liquids. However, she complained of dysphagia to solids since her admission to the hospital and a feeling of food getting stuck in the lower part of the esophagus upon swallowing, requiring retching to induce vomiting. There was no complaint of dysphagia prior to this admission. During this admission, the patient underwent an EGD due to persistent dysphagia, which revealed columns of sloughed mucosa in the lower third of the esophagus (Figure 2).

**Figure 3:**
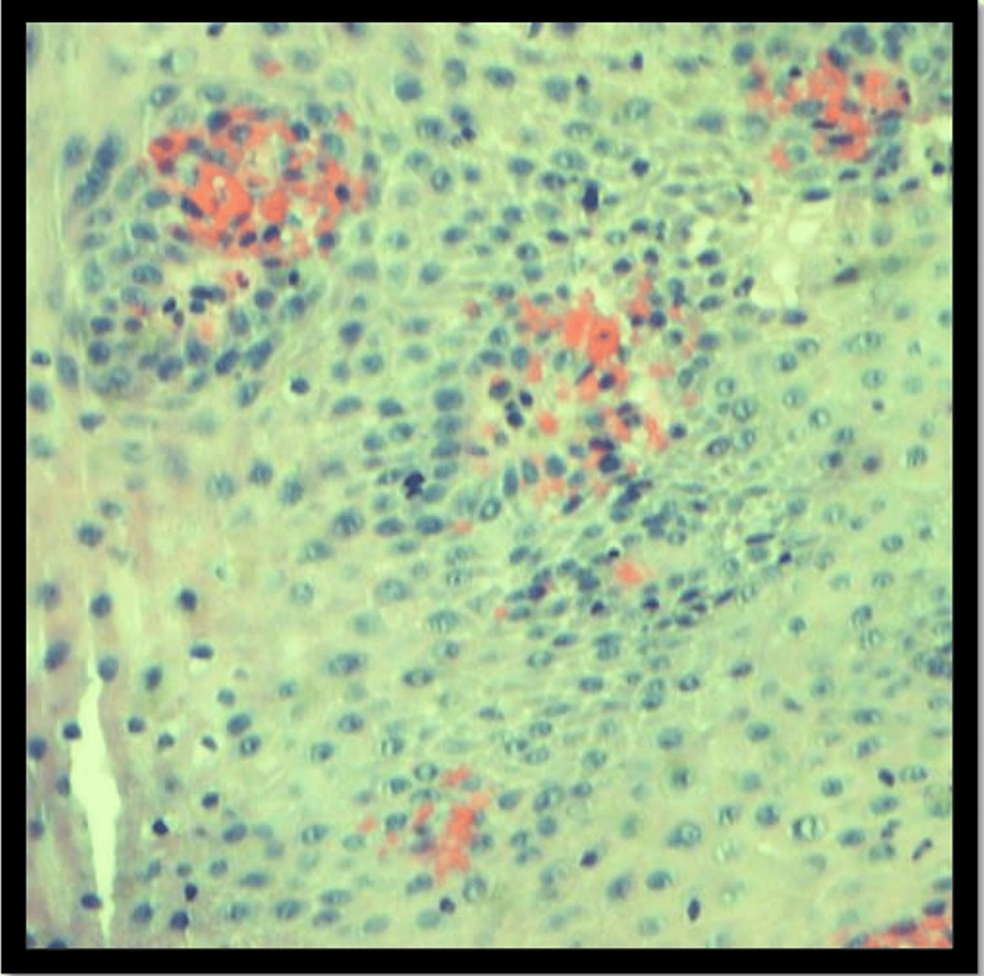
Biopsy revealed fragments of esophageal squamous mucosa showing a few intraepithelial lymphocytes without eosinophils.

A biopsy was performed on the affected area (Figure 3).

**Figure 4:**
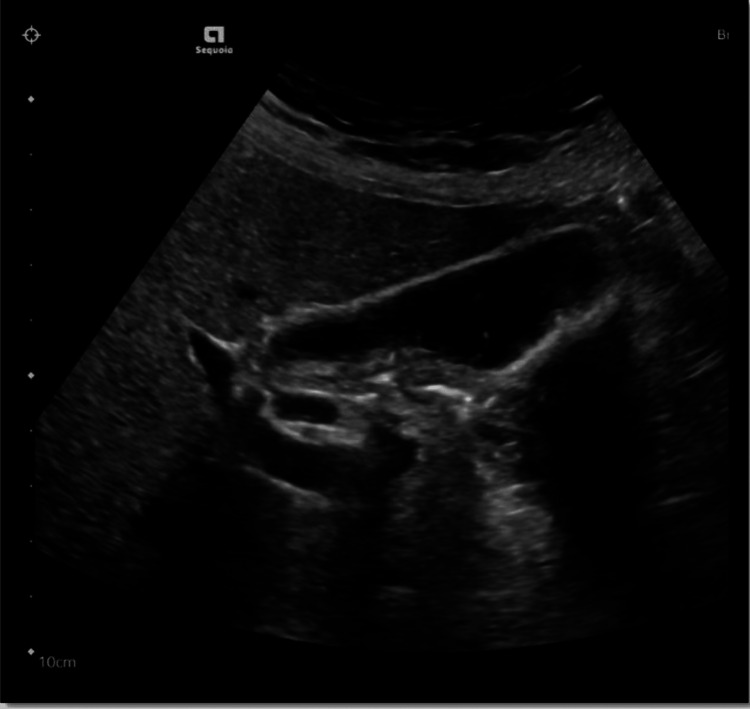
US abdomen: Cholelithiasis. No evidence of acute cholecystitis. US: ultrasound

The patient was prescribed omeprazole 20 mg orally twice daily for a duration of eight weeks. Later a repeat abdominal ultrasound was done which revealed cholelithiasis which was the likely cause of pancreatitis in our patient (Figure 4).

She was discharged following the improvement of her symptoms, with a scheduled close follow-up appointment as an outpatient.

## Discussion

In 1892, Rosenberg used the term esophagitis dissecans superficials (EDS) to describe an endoscopic observation marked by the shedding of significant portions of epithelium [6]. Due to its often-nonspecific symptoms, EDS can easily be misdiagnosed or overlooked. The development of EDS can be attributed to several factors, including idiopathic causes, consumption of hot beverages, exposure to chemical irritants, malignancy, esophageal trauma, the use of medications such as bisphosphonates, nonsteroidal anti-inflammatory drugs, psychoactive medications like SSRIs or SNRIs and methotrexate, and potassium chloride, the presence of celiac disease, collagen disorders, and autoimmune bullous dermatoses [7]. There is a higher prevalence of EDS in patients with multiple underlying health conditions, and a study revealed that 77% of EDS patients are taking five or more medications. This suggests a potential link between EDS and chronic debilitation, indicating an association between the two [4]. Although EDS has been associated with different autoimmune conditions such as celiac disease, lupus, pemphigus vulgaris, bullous pemphigoid, and Stevens-Johnson syndrome, the exact cause of EDS is still not understood [8]. However, cases have been reported with the skin manifestation of systemic lupus erythematosus (SLE) called Bullous systemic lupus erythematosus. The signs and symptoms in patients with bullous SLE include multiple blistering pruritic skin lesions involving the face and trunk, a photosensitive rash over the face and neck, swelling of the right neck lymph node, and joint pain involving her elbows and wrist, and diagnosis is by biopsy [9]. In our patient, the underlying SLE could be a risk factor for her development of EDS. This is a rare occurrence, and limited data is available on this. The clinical presentation of EDS is not consistent or uniform. It can range from incidental findings during endoscopy to esophageal symptoms like dysphagia, heartburn, odynophagia, regurgitation, hematemesis, and melena. Some extreme cases have even reported patients vomiting mucosal casts [10,11]. Our patient was presented with complaints of dysphagia only. EDS is commonly seen in the distal and middle third of the esophagus; it has the potential to affect the entire length of the esophagus [12]. The diagnosis of EDS is by endoscopy, which reveals the characteristic appearance of sloughed mucosal fragments, along with the biopsy to confirm the diagnosis and rule out other possible conditions. It has been proposed that fulfilling three of the following endoscopic criteria indicates the presence of EDS (1) the presence of sloughed esophageal mucosa strips measuring over 2cm in length; (2) normal underlying esophageal mucosa; and (3) the absence of ulcerations or friability in the immediately adjacent esophageal mucosa [13]. Some authors have suggested conducting a subsequent endoscopy after eight weeks, but insufficient evidence substantiates its potential advantages [14]. Endoscopy plays a crucial role in distinguishing EDS from other conditions such as squamous cell carcinoma, candida esophagitis, peptic esophagitis, eosinophilic esophagitis, gastroesophageal reflux disease (GERD), and autoimmune bullous dermatoses [15]. In patients presenting with upper GI symptoms, particularly hematemesis, and showing signs of esophagitis during endoscopy, it is essential to include EDS as a potential diagnosis in the differential diagnosis [16]. Our patient underwent EDG, which was diagnostic of EDS. The histopathological findings of EDS typically include sloughed superficial mucosa, inflammatory infiltrates, and evidence of epithelial regeneration. Additionally, there may be signs of chronic inflammation, such as lymphocytic infiltrates and dilated blood vessels [15]. The histopathological findings of our patient include fragments of esophageal squamous mucosa showing a few intraepithelial lymphocytes without eosinophils. The treatment of EDS focuses on managing symptoms and addressing underlying factors contributing to the condition. In most cases, the shedding of the esophageal mucosa is temporary and resolves on its own without causing any long-term complications. A combination approach involving acid suppression using proton pump inhibitors (PPIs) and discontinuation of the causative medications has been reported to promote healing in cases of EDS. Our patient was treated with omeprazole (40 mg) daily for eight weeks. She responded positively to the treatment, experiencing significant improvement in her clinical symptoms. In cases where the underlying cause of EDS is autoimmune, such as bullous pemphigoid, the use of steroids can be beneficial [17]. A study conducted by Hart et al. revealed that 85.7% of EDS patients who received follow-up endoscopy exhibited improvement and clearance of the sloughed membranes. Regarding prognosis in general, EDS is characterized as a self-limiting condition and often resolves spontaneously without significant long-term complications including stricture formation, GI bleeding, and aspiration pneumonia. The prognosis is generally favorable with proper treatment, which typically involves using medications like PPIs to reduce acid production and discontinuing any medications that may contribute to the condition. In many cases, healing of the esophageal mucosal lining and symptom improvement can be achieved. However, the prognosis may be influenced by factors such as the extent of esophageal damage, underlying medical conditions, and individual response to treatment. Regular monitoring and follow-up with a healthcare professional are essential to track the progress of EDS and ensure appropriate management. Endoscopists should be vigilant about identifying EDS, a rare and harmless condition, to avoid confusing it with other entities like esophagitis or squamous cell carcinoma.

## Conclusions

EDS is a rare and non-malignant condition that endoscopists should be mindful of as this can be easily overlooked and missed. The diagnosis is established through biopsy samples. While there have been reports linking EDS to medications, skin disorders, excessive smoking, and physical injuries, the underlying cause of EDS remains unclear. Despite being a rare condition, it is essential to identify it early to avoid misdiagnosis and unnecessary invasive procedures. Treatment focuses on addressing underlying factors and providing symptomatic relief. Further research is warranted to understand the pathogenesis of EDS better and to establish standardized diagnostic criteria and treatment guidelines.

## References

[1] Hokama A, Yamamoto Y, Taira K (2010). Esophagitis dissecans superficialis and autoimmune bullous dermatoses: a review. World J Gastrointest Endosc.

[2] Senyondo G, Khan A, Malik F, Oranu A (2022). Esophagitis dissecans superficialis: a frequently missed and rarely reported diagnosis. Cureus.

[3] Akhondi H (2014). Sloughing esophagitis: a not so common entity. Int J Biomed Sci.

[4] Purdy JK, Appelman HD, McKenna BJ (2012). Sloughing esophagitis is associated with chronic debilitation and medications that injure the esophageal mucosa. Mod Pathol.

[5] Then EO, Grantham T, Lopez M, Reddy M, Gaduputi V (2021). Esophagitis dissecans superficialis (EDS) secondary to hair dye ingestion: case report and literature review. Clin Pract.

[6] Salehi AM, Salehi H, Hasanzarrini M (2022). Esophagitis dissecans superficialis after COVID-19; a case report. Middle East J Dig Dis.

[7] Hage-Nassar G, Rotterdam H, Frank D, Green PH (2003). Esophagitis dissecans superficialis associated with celiac disease. Gastrointest Endosc.

[8] Carmack SW, Vemulapalli R, Spechler SJ, Genta RM (2009). Esophagitis dissecans superficialis ("sloughing esophagitis"): a clinicopathologic study of 12 cases. Am J Surg Pathol.

[9] Yogarajah M, Sivasambu B, Jaffe EA (2015). Bullous systemic lupus erythematosus associated with esophagitis dissecans superficialis. Case Rep Rheumatol.

[10] Stevens A, Dove GAW (1960). Esophageal cast: esophagitis dissecans superficialis. Lancet.

[11] Kaplan RP, Touloukian J, Ahmed AR (1981). Esophagitis dissecans superficialis associated with pemphigus vulgaris. J Am Acad Dermatol.

[12] Nasir UM, Rodgers B, Panchal D, Choi C, Ahmed S, Ahlawat S (2020). Ferrous sulfate-induced esophageal injury leading to esophagitis dissecans superficialis. Case Rep Gastroenterol.

[13] Johnston DN, Veettil R (2020). Oesophagitis dissecans superficialis - an unusual endoscopic finding. Ulster Med J.

[14] Rokkam VR, Aggarwal A, Taleban S (2020). Esophagitis dissecans superficialis: malign appearance of a benign pathology. Cureus.

[15] Hart PA, Romano RC, Moreira RK, Ravi K, Sweetser S (2015). Esophagitis dissecans superficialis: clinical, endoscopic, and histologic features. Dig Dis Sci.

[16] Orosz E, Patel AV (2021). Sloughing mucosa in esophagitis dissecans superficialis. Clin Gastroenterol Hepatol.

[17] Jaben I, Schatz R, Willner I (2019). The clinical course and management of severe esophagitis dissecans superficialis: a case report. J Investig Med High Impact Case Rep.

